# A case of McLeod syndrome caused by a nonsense variation c.942G>A in the *XK* gene: A case report

**DOI:** 10.3389/fgene.2023.1073139

**Published:** 2023-02-01

**Authors:** Yanling Ying, Shifang Yu, Jingjing Zhang, Ji He, Xianguo Xu, Xiaozhen Hong, Faming Zhu

**Affiliations:** ^1^ Blood Center of Zhejiang Province, Hangzhou, Zhejiang, China; ^2^ Key Laboratory of Blood Safety Research of Zhejiang Province, Hangzhou, Zhejiang, China; ^3^ The Second Affiliated Hospital of Zhejiang University School of Medicine, Zhejiang University, Hangzhou, Zhejiang, China

**Keywords:** McLeod syndrome, *XK* gene, nonsense variation, case report, genotyping

## Abstract

McLeod syndrome is a rare *XK* gene-related progressive, debilitating disease involving multiple systems. The blood group phenotypes in McLeod syndrome patients usually display the Kx antigen loss and a decrease in the Kell blood group system antigen expression. This paper describes a 41-year-old male Chinese patient with McLeod syndrome. He first attended a hospital in 2015 and developed progressively worsening symptoms 4 years ago. As the disease progressed, the patient exhibited memory loss, unresponsiveness, and chorea and displayed elevated creatine kinase levels. However, McLeod syndrome could not be diagnosed by these signs and laboratory results. The patient was readmitted to the hospital in 2020 and was suspected of having McLeod syndrome. Serological analysis of the Kell blood group system and genotyping for the XK blood group system were performed, revealing the weak expression of the K antigen and the negative K antigen. Sequencing of the coding region of the *XK* gene showed a hemizygous c.942G>A variation in the *XK* gene, which resulted in a premature stop codon at position 314 (p.Trp314Ter). Therefore, the patient was diagnosed with McLeod syndrome. In conclusion, this paper presents a case of McLeod syndrome caused by a nonsense variation c.942G>A in the *XK* gene. The analysis of the *XK* gene and blood group antigen is helpful for the diagnosis of McLeod syndrome and for distinguishing it from many other diseases.

## Introduction

**FIGURE 1:**
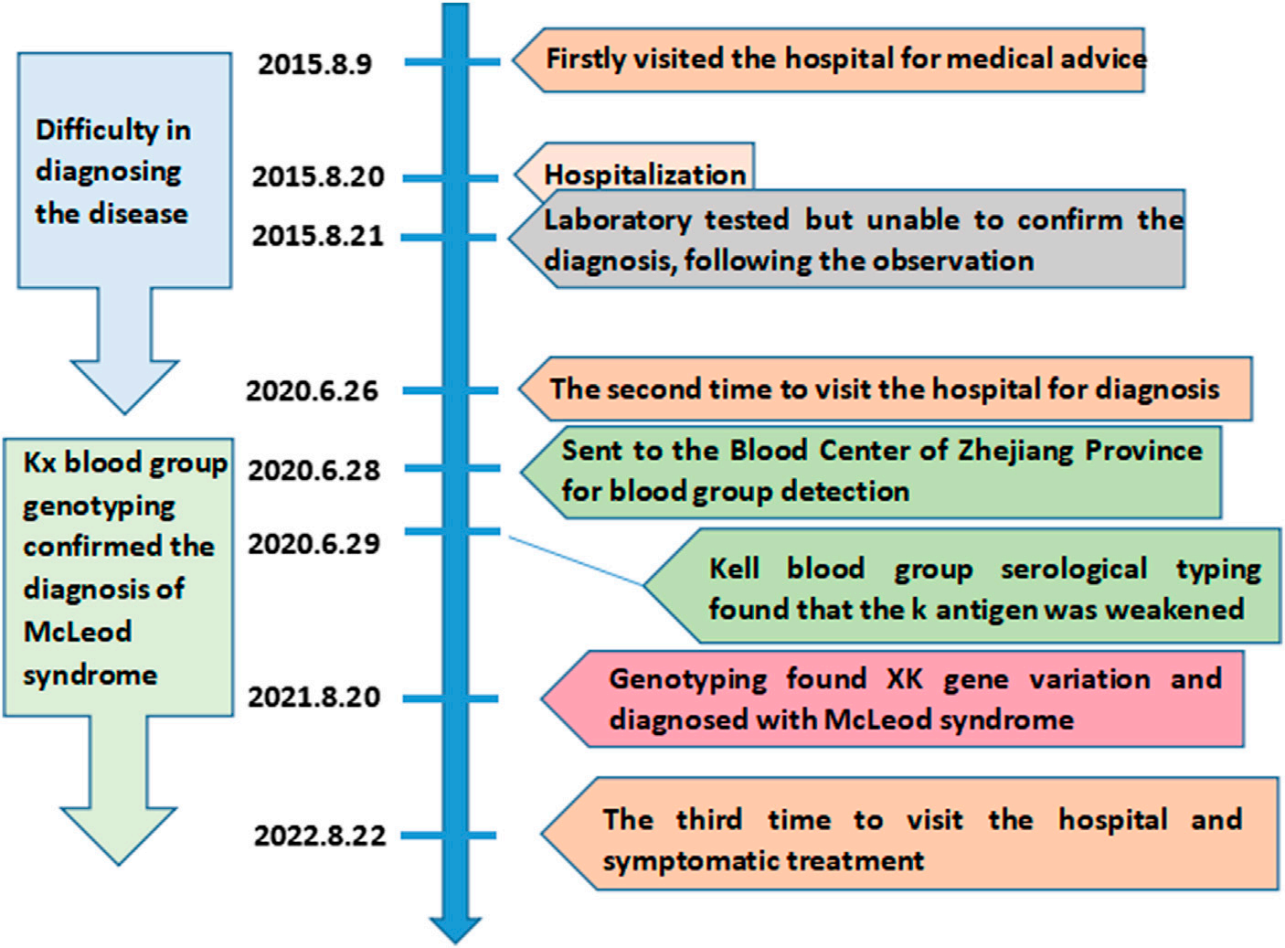
Timeline of the patient’s medical history.

McLeod syndrome is an extremely rare and progressively debilitating disease involving the blood system and neuromuscular and central nervous systems. The clinical symptoms of McLeod syndrome are diverse, including a series of symptoms such as dyskinesia, confusion, acanthocytosis, and elevated muscle creatinine kinase levels (Jung et al., 2007; Walker et al., 2011; Deutschländer et al., 2022). McLeod syndrome is related to the human XK blood group system, which is encoded by the *XK* gene (Roulis et al., 2018). The *XK* gene contains three exons, which encode the XK protein with multiple transmembrane structures, forming the only antigen of the human XK blood group system. The XK protein is linked to the Kell glycoprotein by a single disulfide bond (Kell^Cys72^–XK^Cys347^) (Russo et al., 1998). The absence of the XK protein caused by *XK* gene mutation leads to a decrease in antigen expression of the Kell blood group system on the red blood cell membrane (Balint and Lang, 2020; Chang et al., 2021). Therefore, the red blood cells of McLeod syndrome patients lack the Kx antigen, accompanied by a severe decrease in the Kell antigen expression, which is called the McLeod phenotype (Floch et al., 2021). As the *XK* gene is located on the X chromosome, McLeod syndrome is usually found in male carriers (Murakami et al., 2019). The condition can easily be confused with extrapyramidal diseases due to its rarity and high heterogeneity in clinical symptoms. The patients often require repeated medical attention over a long period of time before achieving a definitive diagnosis. The *XK* variant is a highly specific diagnostic marker for McLeod syndrome (Gassner et al., 2017). Thus, the detection of the *XK* gene variant is the “gold standard” for the diagnosis of McLeod syndrome. Early diagnosis of McLeod syndrome is crucial for treatment. In addition, the lack of the Kx antigen may lead to the allosensitization and hemolytic transfusion reaction when considering blood transfusion. This paper reports a rare case of McLeod syndrome with an XK*N.47 allele for the first time in the Chinese population. Figure 1 depicts the timeline of the patient’s medical history and course of care. This case report was prepared following the CARE guidelines (Riley et al., 2017).

## Case presentation

A 41-year-old Chinese male patient was presented to the Second Affiliated Hospital of Zhejiang University, China, on 9 August 2015, due to a progressively unstable gait for 4 years. The patient reported an unstable gait, weakness in his legs, and involuntary forward bending of both knees at age 37, which had progressively worsened over the past 4 years. Upon inquiry about the medical history, the patient exhibited involuntary limb twisting and had poor movement during childhood. Over the past 2 years, the patient suffered from a decline in memory and slow reactions, resulting in the inability to work. The patient denied any history of other chronic diseases or any similar family history. After admission, the physical examination showed no change in the muscle tone of the limbs. The Hoffman’s sign, Romberg sign, finger-to-nose test, and bilateral Babinski sign were negative. The patient demonstrated slow alternate motions and decreased tendon reflexes in the four limbs. The muscle strength of the four limbs was graded as five. Laboratory examinations revealed that the creatine kinase level was elevated to 4238U/L (standard: <171U/L), with glutamic-oxaloacetic transaminase 106U/L (standard: <35U/L) and lactate dehydrogenase 317U/L (standard: <248U/L). There were no obvious abnormalities in blood coagulation, blood lipids, rheumatism, tumor markers, and glycosylated hemoglobin. The muscle biopsy revealed muscle fiber degeneration. However, these results did not yield a clear diagnosis, and no clinical drug treatment was initiated in 2015. The patient was admitted to the hospital for the second time on 26 June 2020, for further investigation. The patient exhibited poorer movement stability and muscle control. The creatine kinase level rose to 4469U/L (standard: <171U/L), with creatine kinase-MB 99U/L (standard: <48U/L) and lactate dehydrogenase 419U/L (standard: <248U/L). The patient was then suspected of having McLeod syndrome. Thus, blood samples were sent to the Blood Center of Zhejiang Province, China, for the Kell blood group system antigen and *XK* gene analysis.

**FIGURE 2:**
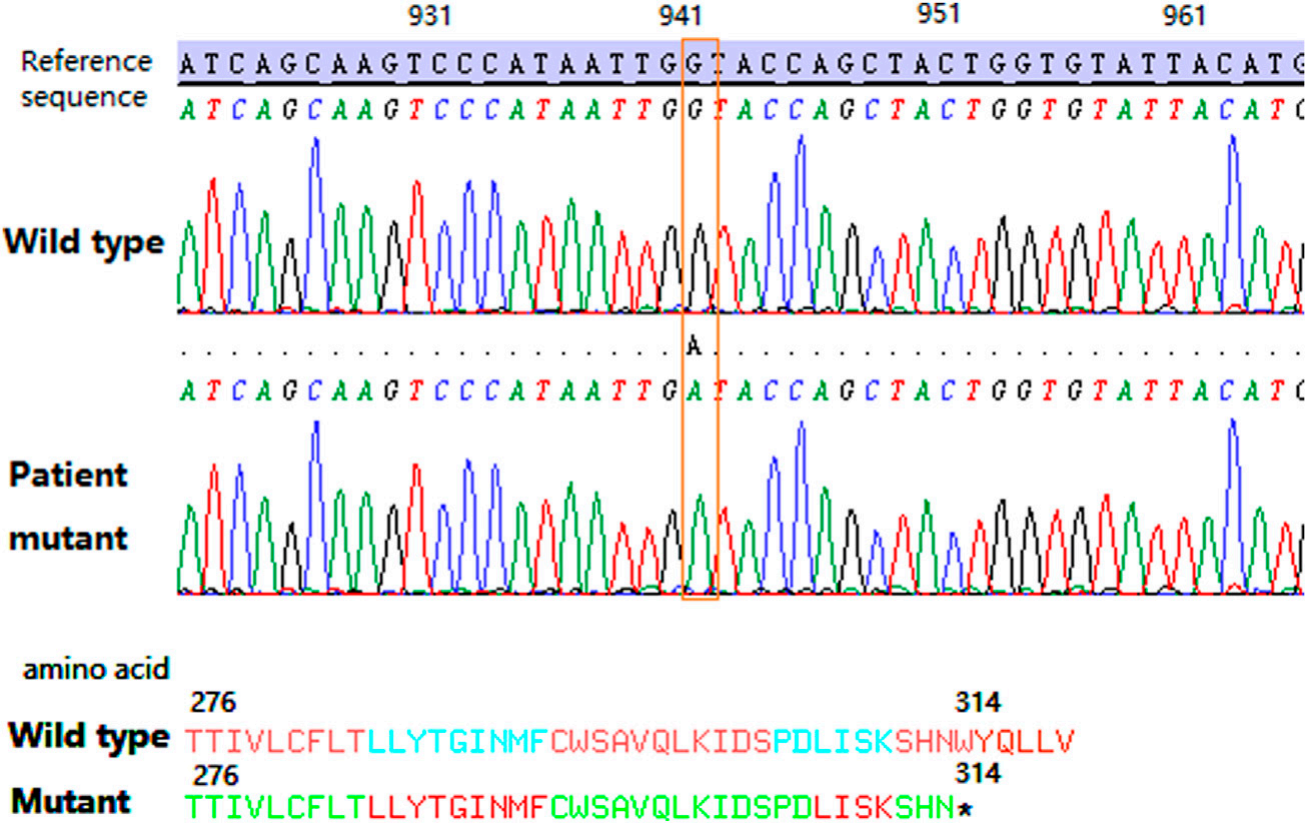
Partial sequencing map of the patient’s *XK* gene. The orange dotted box shows the c.942G>A variation. The asterisk shows the tryptophan change to a stop codon located at the 314 position of the XK protein.

**FIGURE 3:**
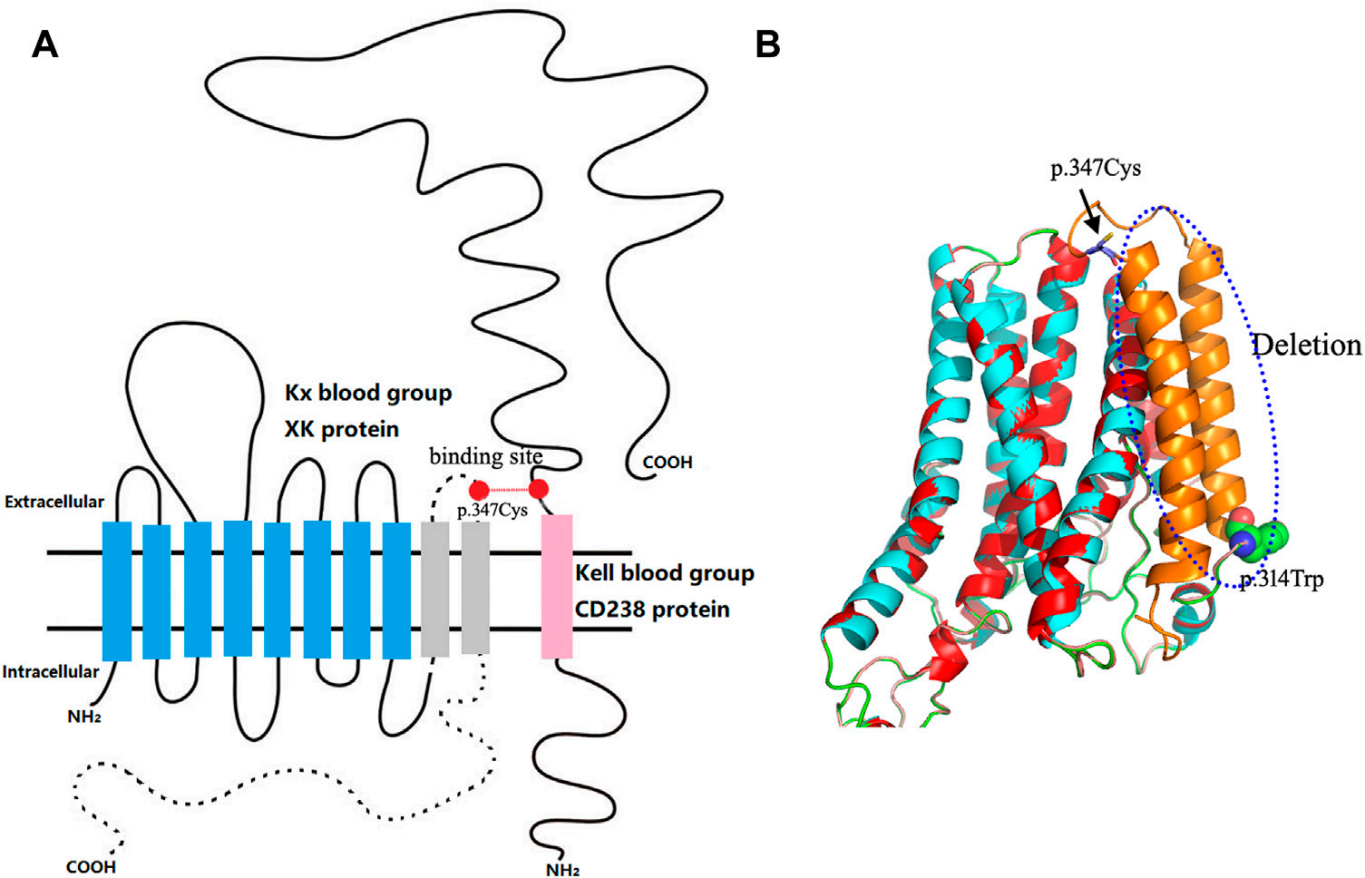
Structural analysis of the patient’s truncated XK protein. **(A)** Schematic diagram of the 10-transmembrane structure of the XK protein. The blue column indicates the retained transmembrane structure of the truncated XK protein; the dotted line and gray column represent deleted structures of the truncated protein, including the last two transmembrane structures, the last extracellular domain, and the entire C-terminal intracellular peptide segment. The red dots are binding sites XK^Cys347^–Kell^Cys72^. This schematic representation is based on the base model constructed by Roulis et al. (2018). **(B)** Comparison of 3D conformations of mutant and wild-type XK proteins. The red and blue areas represent the overlapping regions; the blue dashed box is the deleted structure; the globular amino acid structure represents the truncation point of the protein. The rod-shaped amino acid indicated by the black arrow corresponds to the missing binding site of the Kell blood group system.

For the serological typing of Kell blood group system antigens, red blood cells (RBCs) showed no agglutination with the anti-K antibody (IgM) by the saline tube test at room temperature (Roback et al., 2008). RBCs with the anti-K monoclonal antibody (IgG), anti-Kp^a^ (IgG), and anti-Kp^b^ (IgG) were shown negative by the microcolumn gel card method, and only weak agglutination intensity (2+) was observed in RBCs with anti-k monoclonal antibodies (IgG). Therefore, the serological phenotype of the Kell blood group showed a weak K antigen expression. Unfortunately, the Kx antigen expression was not tested due to the lack of antibodies for the XK blood group system. Subsequently, the coding region sequence of exons 1–3 in the *XK* gene was amplified by a polymerase chain reaction in our laboratory. Amplicons were purified by enzyme digestion and then directly sequenced and analyzed. The sequencing results showed that the patient carried a hemizygous c.942G>A variation in the *XK* gene, corresponding to the XK*N.47 allele in the International Society of Blood Transfusion (ISBT) blood group database (Figure 2). This variation leads to a premature stop codon at position 314 and results in an incomplete C-terminally truncated protein (p.Trp314Ter) (Figure 2). The nucleotide sequence of the allele has been submitted to the GenBank database with the accession number OK18693. Moreover, the protein structure was simulated and analyzed with PyMOL software. The result showed that the incomplete XK protein lacked the binding site for the Kell antigen (Figure 3). The results clarified the molecular basis of the *XK* gene, and a definitive diagnosis of McLeod syndrome was confirmed.

The patient was admitted for the third time on 22 August 2022, due to hepatic insufficiency. The physical examination was the same as before, while electromyography showed peripheral nerve damage. Unfortunately, there is, currently, no effective cure for the disease. Consequently, the patient was given symptomatic treatment, including the ganglioside, coenzyme Q10, vitamin E, and vitamin B2, which promoted nerve repair. The patient was discharged from the hospital in good spirits. Although he still complained of an unsteady gait, the condition showed no worsening.

## Discussion

In this case report, McLeod syndrome was confirmed based on the full coding region sequence analysis of the *XK* gene. The incidence of McLeod syndrome is extremely low, with only hundreds of patients worldwide. There are no epidemiological data on the incidence of this disease (Urata et al., 2019). In China, the first case of McLeod syndrome was reported in 2013 (Man et al., 2013). Up to now, only a few cases have been reported in China (Man et al., 2013; Wu et al., 2019; Yu et al., 2021). The complexity and heterogeneity of the symptoms, lack of knowledge, and specific laboratory diagnostic methods for McLeod syndrome diagnosis hinder its early detection.

McLeod syndrome is characterized by variations in the *XK* gene (Jung et al., 2003; Arnaud et al., 2009; Srikanth et al., 2020). At present, 48 kinds of variations in the *XK* gene have been identified and described in the ISBT blood group database. The variations mainly include nucleotide deletions/insertions, nucleotide point mutations, and changes in transcriptional splicing sites. These variations usually lead to the premature termination of the protein translation or alternative splicing of the transcription process, resulting in the deletion or shortening of the XK protein that carries Kx antigens (Arnaud et al., 2009). Alternatively, *XK* variants may also arise by large deletions, which affect telomeric and centromeric neighbor genes that dominate the clinical phenotype (Roulis et al., 2018). In this case, the c.942G>A variation located in exon 3 of the *XK* gene caused the premature termination of the protein. The mutant protein lost the last two transmembrane structures, the last extracellular domain, and the entire C-terminal intracellular peptide segment, thus affecting the protein function *in silico*. The structural analysis of mutant proteins revealed the effects of variations in the *XK* gene on the protein function. Meanwhile, the binding site between the XK and KEL proteins was omitted in the truncated protein, affecting the expression of Kell blood group antigens. The patient demonstrated a typical McLeod red blood cell phenotype and genotype.

This is a rare case with a nonsense mutation of the *XK* gene. The allele XK*N.47 (c.942G>A) was previously reported in the ISBT database (Floch et al., 2021). Furthermore, this pathogenic variation of McLeod syndrome was reported for the first time in the Chinese population. Previously, Supple et al. (2001) reported the same *XK* gene mutation (c.941G>A, p.Trp314Ter) in a patient with McLeod syndrome. These two variations were located in the same codon, and both of them formed a stop codon, which led to aberrant protein formation. Various mutation types also result in different allele variants. Clinical phenotypes of nonsense or frameshift mutations are generally predicted to be more harmful than those of missense mutations. Nonsense variations c.942G>A and c.941G>A formed a stop codon in the *XK* gene, impairing the neuromuscular or cerebral function, while missense mutations (c.746C>G, p.Arg222Gly; c.1061G>A, p.Glu327Lys) (Russo et al., 2002; Jung et al., 2003) only affected the McLeod hematologic phenotype.

Sequential analysis of the *XK* gene is a highly specific diagnostic marker for McLeod syndrome (Gassner et al., 2017). Previous studies have investigated the importance of early and accurate diagnosis for McLeod syndrome, outlining the crucial role of hematology analyses in early detection (Kelly et al., 2022). The *XK* gene sequence analysis could be used for the early diagnosis and symptomatic treatment of the disease, especially in elderly male patients presenting with clinical symptoms similar to chorea. This patient’s disease showed a slow and steady progression. Unfortunately, no specific drugs are available to cure the disease caused by genetic mutations, even if the diagnosis is clear. However, genetic analysis plays a central role in the diagnosis and could improve the patient’s state of mind. In addition, the identified allele variant of XK allows the assessment of family members at risk. Furthermore, the diagnosis provides a basis for follow-up research on treatment measures. It is also worth noting that patients with the McLeod phenotype lack the public red cell antigen Kx, which poses a high risk for the formation of anti-public alloantibodies and the acute hemolytic transfusion reaction if incompatible red blood cell concentrates are administered.

In conclusion, this paper reports a rare case of McLeod syndrome caused by the premature termination of protein translation due to a c.942G>A variation in the *XK* gene. Routine sequencing for the *XK* gene is recommended for male patients with chorea-like clinical symptoms, which will help in the early diagnosis of McLeod syndrome.

## References

[B1] Arnaud L. Salachas F. Lucien N. Maisonobe T. Le Pennec P. Y. Babinet J. (2009). Identification and characterization of a novel XK splice site mutation in a patient with McLeod syndrome. Transfusion 49, 479–484. 10.1111/j.1537-2995.2008.02003.x 19040496

[B2] Balint B. Lang A. E. (2020). Expert comment to: Novel Xp21.1 deletion associated with unusual features in large McLeod syndrome kindred. Park. Relat. Disord. 79, 133–134. 10.1016/j.parkreldis.2019.02.024 30858069

[B3] Chang K. Y. Chang C. K. Lin M. H. Yang C. C. Lo S. C. (2021). Novel c.435delC mutation in XK gene found in a Taiwanese patient with McLeod syndrome. Transfusion 61, E28–E30. 10.1111/trf.16316 33569764

[B4] Deutschländer A. B. Dickson D. W. Wszolek Z. K. (2022). Neuropathology of McLeod syndrome. Mov. Disord. 37, 644–646. 10.1002/mds.28882 34913196

[B5] Floch A. Lomas-Francis C. Vege S. Westhoff C. M. (2021). Three new XK alleles; two associated with a McLeod RBC phenotype. Transfusion 61, E69–E70. 10.1111/trf.16650 34487382

[B7] Gassner C. Brönnimann C. Merki Y. Mattle-Greminger M. P. Sigurdardottir S. Meyer E. (2017). Stepwise partitioning of Xp21: A profiling method for XK deletions causative of the McLeod syndrome. Transfusion 57, 2125–2135. 10.1111/trf.14172 28555782

[B8] Jung H. H. Danek A. Frey B. M. (2007). McLeod syndrome: A neurohaematological disorder. Vox Sang. 93, 112–121. 10.1111/j.1423-0410.2007.00949.x 17683354

[B9] Jung H. H. Hergersberg M. Vogt M. Pahnke J. Treyer V. Röthlisberger B. (2003). McLeod phenotype associated with a XK missense mutation without hematologic, neuromuscular, or cerebral involvement. Transfusion 43, 928–938. 10.1046/j.1537-2995.2003.t01-1-00434.x 12823753

[B10] Kelly K. Helander L. Hazegh K. Stanley C. Moss R. Mack S. (2022). Cryopreservation of rare pediatric red blood cells for support following bone marrow transplant. Transfusion 62 (5), 954–960. 10.1111/trf.16878 35403731

[B11] Man B. L. Yuen Y. P. Yip S. F. Ng S. H. (2013). The first case report of McLeod syndrome in a Chinese patient. BMJ Case Rep. 2013, bcr2013200205. 10.1136/bcr-2013-200205 PMC3762422 23943810

[B12] Murakami T. Abe D. Matsumoto H. Tokimura R. Abe M. Tiksnadi A. (2019). A patient with McLeod syndrome showing involvement of the central sensorimotor tracts for the legs. BMC Neurol. 19, 301. 10.1186/s12883-019-1526-9 31775676 PMC6882147

[B13] Riley D. S. Barber M. S. Kienle G. S. Aronson J. K. von Schoen-Angerer T. Tugwell P. (2017). CARE guidelines for case reports: Explanation and elaboration document. J. Clin. Epidemiol. 89, 218–235. 10.1016/j.jclinepi.2017.04.026 28529185

[B14] Roback J. D. Raecombs M. Grossman B. J. (2008). AABB technical manual[M]. 16th ed Betheda: AABB Press, 874–876.

[B15] Roulis E. Hyland C. Flower R. Gassner C. Jung H. H. Frey B. M. (2018). Molecular basis and clinical overview of McLeod syndrome compared with other neuroacanthocytosis syndromes: A review. JAMA Neurol. 75, 1554–1562. 10.1001/jamaneurol.2018.2166 30128557

[B16] Russo D. Redman C. Lee S. (1998). Association of XK and Kell blood group proteins. J. Biol. Chem. 273, 13950–13956. 10.1074/jbc.273.22.13950 9593744

[B17] Russo D. C. Lee S. Reid M. E. Redman C. M. (2002). Point mutations causing the McLeod phenotype. Transfusion 42 (3), 287–293. 10.1046/j.1537-2995.2002.00049.x 11961232

[B18] Srikanth P. Al-Louzi O. A. Bowley M. P. Videnovic A. (2020). A novel XK gene mutation causative of McLeod syndrome. Mov. Disord. Clin. Pract. 7, 340–342. 10.1002/mdc3.12912 32258238 PMC7111571

[B19] Supple S. G. Iland H. J. Barnett M. H. Pollard J. D. (2001). A spontaneous novel XK gene mutation in a patient with McLeod syndrome. Br. J. Haematol. 115 (2), 369–372. 10.1046/j.1365-2141.2001.03121.x 11703337

[B20] Urata Y. Nakamura M. Sasaki N. Shiokawa N. Nishida Y. Arai K. (2019). Novel pathogenic XK mutations in McLeod syndrome and interaction between XK protein and chorein. Neurol. Genet. 5, e328. 10.1212/NXG.0000000000000328 31086825 PMC6481271

[B21] Walker R. H. Jung H. H. Danek A. (2011). Neuroacanthocytosis. Handb. Clin. Neurol. 100, 141–151. 10.1016/B978-0-444-52014-2.00007-0 21496574

[B22] Wu J. Lu A. D. Zhang L. P. Zuo Y. X. Jia Y. P. (2019). Study of clinical outcome and prognosis in pediatric core binding factor-acute myeloid leukemia. Zhonghua Xue Ye Xue Za Zhi 40, 52–57. 10.3760/cma.j.issn.0253-2727.2019.01.010 30704229 PMC7351698

[B23] Yu Z. Fu Y. Fan D. S. (2021). A case report of O'Sullivan-McLeod syndrome. Zhonghua Nei Ke Za Zhi 60, 997–998. 10.3760/cma.j.cn112138-20201120-00956 34689522

